# Transforming growth factor beta-2 drives trabecular meshwork progenitor cell differentiation through SMAD2/3 signaling

**DOI:** 10.1093/stmcls/sxag033

**Published:** 2026-05-26

**Authors:** Xiaochen Fan, Emine K Bilir, Olivia A Kingston, Victoria R Kearns, Colin E Willoughby, Carl M Sheridan

**Affiliations:** Department of Eye and Vision Science, Institute of Life Course and Medical Sciences, University of Liverpool, Liverpool, L69 3BX, United Kingdom; Institute of Ophthalmology, University College London, London, EC1V 9EL, United Kingdom; Department of Eye and Vision Science, Institute of Life Course and Medical Sciences, University of Liverpool, Liverpool, L69 3BX, United Kingdom; Department of Musculoskeletal and Ageing Science, Institute of Life Course and Medical Sciences, University of Liverpool, Liverpool, L69 3BX, United Kingdom; Department of Eye and Vision Science, Institute of Life Course and Medical Sciences, University of Liverpool, Liverpool, L69 3BX, United Kingdom; Department of Eye and Vision Science, Institute of Life Course and Medical Sciences, University of Liverpool, Liverpool, L69 3BX, United Kingdom; Department of Eye and Vision Science, Institute of Life Course and Medical Sciences, University of Liverpool, Liverpool, L69 3BX, United Kingdom; Centre for Genomic Medicine, Biomedical Sciences Research Institute, Ulster University, Coleraine, BT52 1SA, United Kingdom; Department of Eye and Vision Science, Institute of Life Course and Medical Sciences, University of Liverpool, Liverpool, L69 3BX, United Kingdom

**Keywords:** glaucoma, trabecular meshwork, progenitor cells, cell differentiation, TGFβ

## Abstract

Primary open-angle glaucoma (POAG) is a major cause of irreversible blindness, yet its underly mechanisms remain unclear. Elevated intraocular pressure (IOP), the only modifiable risk factor for POAG, arises from increased resistance to aqueous humor outflow within the conventional outflow pathway, which comprises the trabecular meshwork (TM) and the inner wall of Schlemm’s canal. Dysfunction and cellular loss within this pathway, particularly in the TM, are consistent features of the disease; however, the mechanisms responsible for impaired tissue maintenance and regenerative failure remain unclear. TM progenitor cells (TMPCs) have the capacity to replace lost TM cells, suggesting that impaired progenitor function may contribute to disease progression. Transforming growth factor beta 2 (TGFβ2), which is consistently elevated in the aqueous humor of POAG patients, plays a key role in regulating stem cell differentiation. We proposed that excess TGFβ2 disrupts TMPC function, leading to progenitor depletion and TM dysfunction. Here, we show that TGFβ2 drives TMPCs toward a differentiated, fibrotic phenotype, increasing TM and profibrotic gene expression while reducing progenitor markers. These effects are mediated through TGFβ2–SMAD2/3 signaling, as inhibition of this pathway preserves TMPC characteristics and suppresses fibrotic gene induction. Our findings identify TGFβ2–SMAD2/3 signaling as a regulator of TMPC fate in vitro and suggest a potential mechanism by which elevated TGFβ2 may influence TMPC behavior under pathological conditions.

## Graphical abstract

**Graphical Abstract:**
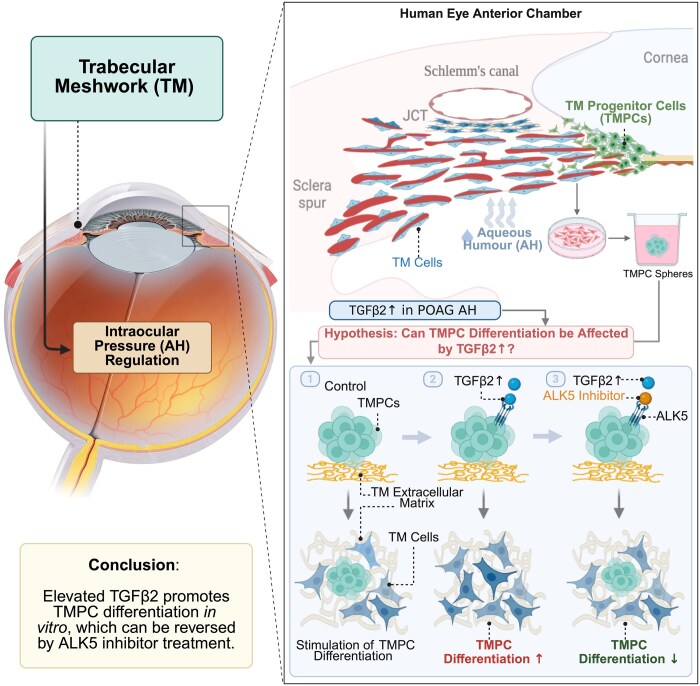

Significance statement Trabecular meshwork (TM) dysfunction drives elevated intraocular pressure in primary open-angle glaucoma, yet mechanisms underlying impaired tissue maintenance remain unclear. TM progenitor cells (TMPCs) may support regeneration, but their regulation is poorly defined. Here, we identify TGFβ2, elevated in glaucoma, as a key regulator of TMPC fate. TGFβ2 promotes TMPC differentiation toward a fibrotic phenotype via SMAD2/3 signaling, with loss of progenitor markers. Inhibition of this pathway preserves TMPC characteristics and suppresses fibrotic gene expression. These findings identify TGFβ2–SMAD2/3 signaling as a potential therapeutic target to preserve TM regeneration in glaucoma.

## Introduction

Glaucoma is the leading cause of irreversible blindness worldwide, with primary open-angle glaucoma (POAG) being its most prevalent form, affecting over 50 million people globally.^1^ POAG is marked by progressive optic nerve degeneration and visual field loss, often linked to elevated intraocular pressure (IOP), the only modifiable risk factor.^2–8^ IOP is maintained by the balance of aqueous humor (AH) production and outflow, which is mainly regulated by the trabecular meshwork (TM) and the inner wall of Schlemm’s canal at the anterior chamber angle.^9^

TM cells are essential for maintaining AH outflow, but their number declines with age and is further significantly reduced in POAG,^10–12^ increasing outflow resistance and impairing IOP control. Resident TM progenitor cells (TMPCs), located mainly in the transitional zone between the TM and corneal endothelium, have the potential to repopulate lost TM cells.^13–16^ These cells express stemness-associated genes such as OCT4, PAX6, and SOX2.^17^^,^^18^ The TMPC population isolated from non-glaucomatous eyes can be expanded in vitro, where they demonstrate the capacity to differentiate into functional TM-like cells.^19^

In the context of aging and glaucoma, studies report a reduction in overall TM cellularity and a decline in stem/progenitor cell content in glaucomatous donor eyes compared to age-matched controls,^20^ although the mechanisms underlying progenitor dysfunction and failed repopulation in vivo remain poorly defined. Elevated transforming growth factor-beta 2 (TGFβ2) levels in the AH of POAG patients^5^^,^^6^^,^^21^ may contribute to this failure, as TGFβ2 has been shown in other progenitor systems to suppress proliferation and promote premature differentiation, leading to stem cell depletion.^22–26^ Within TGFβ2 signaling, the SMAD2/3 and SMAD1/5/8 branches are key regulators of stem cell fate,^27^^,^^28^ often exerting opposing roles with SMAD2/3 promoting differentiation, whereas SMAD1/5/8 supports progenitor maintenance.^29^^,^^30^

Despite these insights, the specific role of TGFβ2 in regulating TMPC differentiation and the signaling mechanisms involved remain unclear. In our previous transcriptomic analysis of TMPCs, TGFβ signaling was identified as a key regulator of TMPC development and differentiation.^31^ Specifically, we observed decreased SMAD2/3 activity alongside increased SMAD1/5/8 signaling, suggesting that SMAD2/3 and SMAD1/5/8 may play opposing roles in the control of TMPC behavior.

Building on these observations, we show that elevated TGFβ2 drives TMPCs toward a more differentiated state. Pharmacological inhibition further identifies the ALK5-SMAD2/3 pathway as the main mediator of this effect, highlighting its role in directing TMPC fate. Together, these findings suggest that increased TGFβ2 signaling may push progenitor cells out of the stem-like state and into differentiation, potentially contributing to their depletion in POAG. Targeting this pathway could therefore help preserve TMPC function and support long-term tissue homeostasis.

## Materials and methods

### TM cell and progenitor cell culture

Human donor corneal rims (*n* = 3; Table S1) were obtained from St Paul’s Eye Unit, Royal Liverpool University Hospital under ethical approval (RETH000833), in accordance with the Declaration of Helsinki. TM tissue was dissected from the corneoscleral rim as described previously^32^ and cultured in low-glucose Dulbecco’s Modified Eagle Medium (DMEM) (Sigma, UK) supplemented with 10% heat-inactivated fetal bovine serum (Biosera, UK), 2 mM L-glutamine, 100U/mL penicillin, and 100 μg/mL streptomycin at 37 °C, 5% CO_2_. TM cells were passaged at ∼80% confluency. Isolated TM cells were characterized by quantifying the myocilin (MYOC) expression after dexamethasone (100 nM, Sigma-Aldrich, USA) treatment for 7 days using immunostaining and western blotting/RT-qPCR (Figure S3).

TMPCs were isolated from TM cultures at passage 5, which represented the earliest passage yielding sufficient cell numbers for reliable progenitor enrichment. For TMPC isolation, 20,000 cells per well were plated in suspension in 24-well plates (Greiner Bio-One, UK) in serum-free DMEM supplemented with 20 ng/mL EGF, 20 ng/mL bFGF (PeproTech, UK), 5 µg/mL heparin sodium (Sigma, UK), and 1× B27 (Thermo Fisher, UK). Growth factor-supplemented medium was refreshed every other day. TMPC spheres were collected on day 7 for downstream applications.

### TMPC differentiation on TM cell-derived extracellular matrix

To generate extracellular matrix (ECM), primary TM cells at passages 7 and 8, derived from the same donors as the TMPCs, were cultured in 24-well plates for 7 days. These later passages were used solely for ECM preparation due to donor tissue limitations. After Phosphate-Buffered Saline (PBS) washing, cells were removed by incubation with 2% ammonium hydroxide (Fisher Scientific, UK) in PBS for 6–10 min, followed by 5 PBS washes (5 min each). ECM-coated plates were stored at −80°C until use. TMPC spheres were then seeded onto matched donor ECM and cultured in serum-free DMEM for 7 days to promote differentiation.

### TGFβ2 treatment during TMPC differentiation

To evaluate the TGFβ2 effect on TMPC differentiation, TMPC spheres were seeded onto ECM, cultured in serum-free DMEM containing 1% penicillin/streptomycin, and treated every other day with 5 ng/mL TGFβ2 (Peprotech, UK), corresponding to concentrations observed in the AH of POAG patients.^21^ Phase-contrast images were captured on days 1, 3, and 5 using a Zeiss microscope to assess cellular growth area. On day 7, cells were fixed or harvested for RNA and protein extraction.

### SMAD pathway and ALK inhibitor studies

Prior to the main experiment investigating the effect of ALK inhibition on TGFβ2-driven TMPC differentiation, optimization studies were performed to determine the most effective concentrations of SMAD pathway inhibitors.

Pilot experiments demonstrated that TGFβ2 stimulation for 30 min robustly activated the SMAD2/3 pathway in primary TM cells, whereas the SMAD1/5/8 pathway was not significantly affected under these conditions. In contrast, in TMPCs, TGFβ2 treatment for 24 h resulted in clear activation of the SMAD1/5/8 pathway. Based on these findings, TGFβ2 (10 ng/mL) treatment for 30 min in primary TM cells was used to evaluate the inhibitory efficiency of SB431542 (ALK5 inhibitor; MERCK, UK) on SMAD2/3 signaling. Similarly, TGFβ2 treatment for 24 h in TMPCs was used to assess the inhibitory efficiency of ML347 (ALK1 inhibitor; MERCK, UK) on SMAD1/5/8 signaling. For both inhibitors, concentrations of 2, 5, 10, and 20 µM were tested.

To determine whether ALK1 or ALK5 inhibition could prevent TGFβ2-induced TMPC differentiation, TMPC spheres derived from 3 donors were pretreated with 5 µM ML347 or SB431542 for 24 h to establish a low basal level of pathway activity. The spheres were subsequently exposed to TGFβ2 (5 ng/mL) for 7 days, with inhibitors replenished every other day to maintain sustained pathway suppression. At the conclusion of treatment, cells were collected for protein analysis, RNA expression studies, and immunocytochemical assessment.

### Quantification of sphere outgrowth

The phase contrast images of the differentiated TM spheres on days 1, 3, and 5 were taken using the ZEISS Axio Vert.A1 Inverted Microscope (ZEISS, UK). The cell outgrowth areas of the differentiated TM spheres were measured using Image J 1.48. The ratio of sphere cell outgrowth area was calculated using the equation: Ratio of sphere cell outgrowth area (day X) = Sphere area (day X)/Sphere area (day 1).

### Immunocytochemistry

Cells (±TGFβ2) were fixed in 10% NBF for 15 min, permeabilized with 1% Triton X-100 for 5 min and blocked at 37 °C for 30 min. Cells were incubated overnight at 4 °C with primary antibodies (Table S2), followed by secondary antibodies (Table S3) for 1 h at 37 °C. Nuclei were counterstained with DAPI. Imaging was performed using a Zeiss Apotome microscope (Zeiss, UK).

### Protein extraction and western blotting

Cells were lysed in RIPA buffer, sonicated, and centrifuged (15 min, full speed).^32^ Protein concentration was quantified by BCA assay (Thermo Fisher, UK). Equal protein (20 µg) was mixed with 2× Laemmli buffer, denatured (95 °C, 5 min), and loaded onto SDS-PAGE gels. After electrophoresis, proteins were transferred to nitrocellulose membranes using the Trans-Blot Turbo Transfer System (Bio-Rad, UK). Membranes were blocked with Odyssey Tris-Buffered Saline (TBS) or 5% Bovine Serum Albumin (BSA) buffer and incubated with primary (Table S4) and secondary antibodies (Table S5). Bands were visualized using the Odyssey Imaging System.

### RNA extraction and RT–qPCR

RNA was extracted using the RNeasy Mini Kit (Qiagen, UK) with on-column DNase I digestion. RNA quality and quantity were assessed via Nanodrop 2000 (Thermo Fisher, UK). cDNA synthesis was performed using the nanoScript2 RT kit (Qiagen, UK). Primers (Eurogentec, UK) were designed using Primer-BLAST and validated via standard and melt curve analysis. Only primer sets with a single melt peak and *R*^2^ > 0.8 were used. RT-qPCR was carried out using Precision PLUS SYBR Green (Primer Design, UK) on the LightCycler^®^ 96 (Roche, UK). Data were normalized to GAPDH gene and analyzed using the 2^–ΔΔCT^ method. Primer and PCR conditions are shown in Tables S6 and S7.

### Bioinformatics analyses

In our earlier study, we identified genes that are expressed differently in primary TM cells, TMPC spheres, and TM cells derived from TMPCs.^18^ In this study, we focused on the genes that change during the transition from TMPCs to differentiated TM cells to better understand TMPC differentiation related differentially expressed genes (DEGs) and pathways. Briefly, the DEGs were uploaded to Galaxy/ProteoRE platform^33^ for predicting the DEGs related to the stem cell differentiation using topGO enrichment analysis. The DEGs from the enriched differentiation-related GO functions related to the TM origin were selected for further pathway analyses (Tables S8). The TM differentiation-related DEGs were uploaded to Cytoscape (v3.10.4)^34^ and the enriched pathways were predicted using ClueGO plugin.^35^ The activation/inhibition of the TGFβ pathway was predicted using the DEGs between TMPCs and TM cells by ingenuity pathway analysis (Qiagen, UK).^36^ Enrichment analysis was performed using a 2-sided hypergeometric test with Bonferroni step-down correction, and pathways with adjusted *P* < .05 were considered significant.

### Statistical analyses

All statistical analyses were conducted using GraphPad Prism (version 8.0.2, USA, www.graphpad.com). For paired comparisons between 2 groups, a 2-tailed *t*-test or Mann–Whitney test was applied as appropriate. Two-way ANOVA was used for multiple-group comparisons. A *P* value < .05 was considered statistically significant.

## Results

### TGFβ-SMAD2/3 pathways play a central role in regulating the TMPC differentiation by bioinformatics analyses

**Figure 1.**
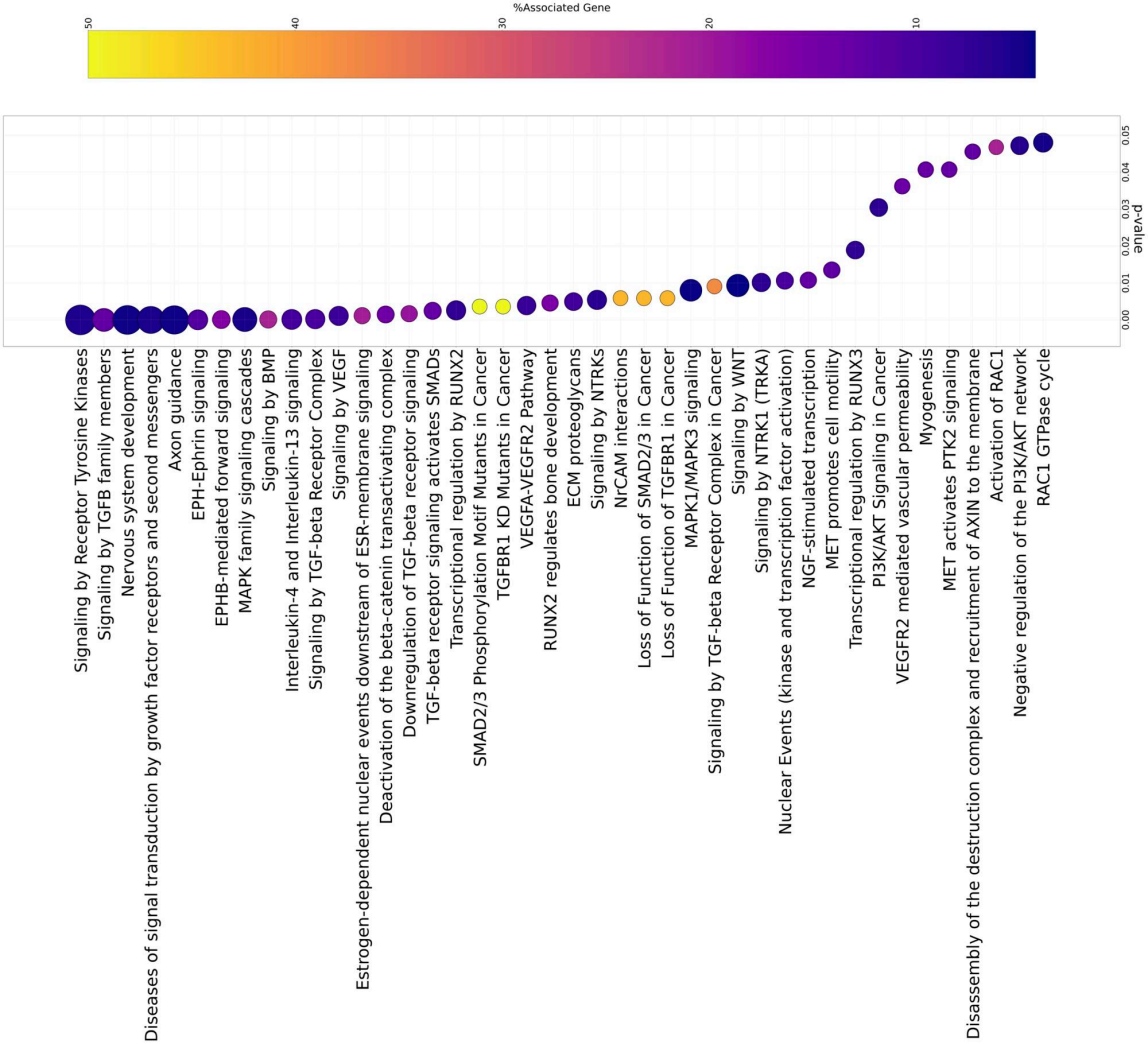
TGFβ signaling pathways are key regulators of trabecular meshwork progenitor cell (TMPC) differentiation. Dot plot showing significantly enriched pathways involved in TMPC differentiation, identified using the ClueGO plugin in Cytoscape. Dot color indicates the proportion of differentially expressed genes (DEGs) (differentiated TM vs TMPCs) within each pathway, and dot size represents the number of DEGs. Statistical significance was determined using a 2-sided hypergeometric test with Bonferroni step-down correction. Pathways with adjusted *P* < .05 were considered significant.

**Figure 2.**
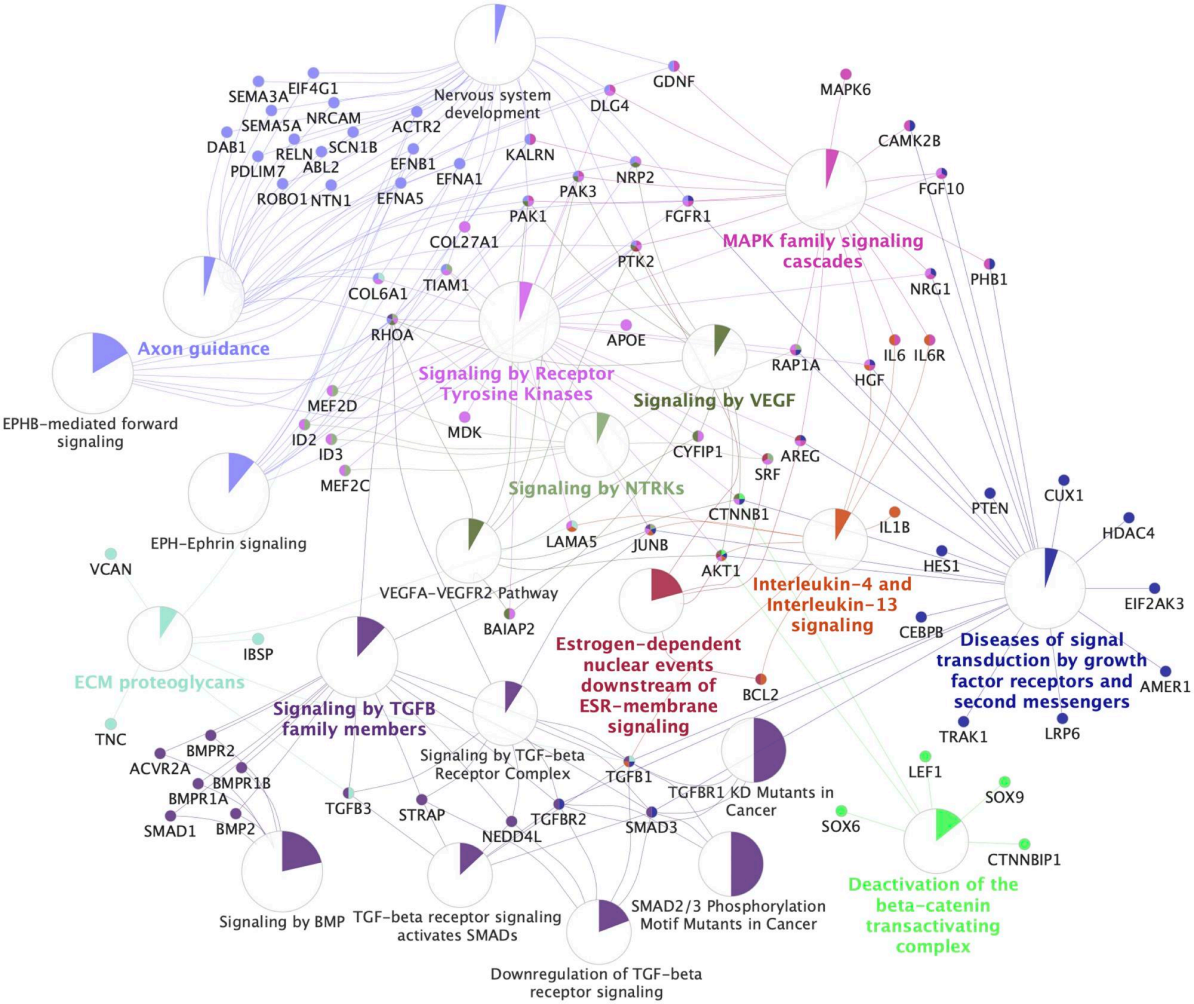
Functional pathway interaction network associated with TMPC differentiation. Pathway interaction network generated using the ClueGO plugin in Cytoscape, showing functionally grouped pathways enriched among DEGs (differentiated TM vs TMPCs). Each central node represents a pathway, displayed as a pie chart showing the proportion of visible genes contributing to that pathway. Central node size represents the significance level of the pathway, and node color segments indicate the pathway group clusters. Peripheral nodes represent the DEGs. Edges represent pathway interrelations based on kappa statistics, indicating the degree of gene overlap between pathways. Enrichment analysis was performed using a 2-sided hypergeometric test with Bonferroni step-down correction, and pathways with adjusted *P* < .05 were considered significant.

**Figure 3.**
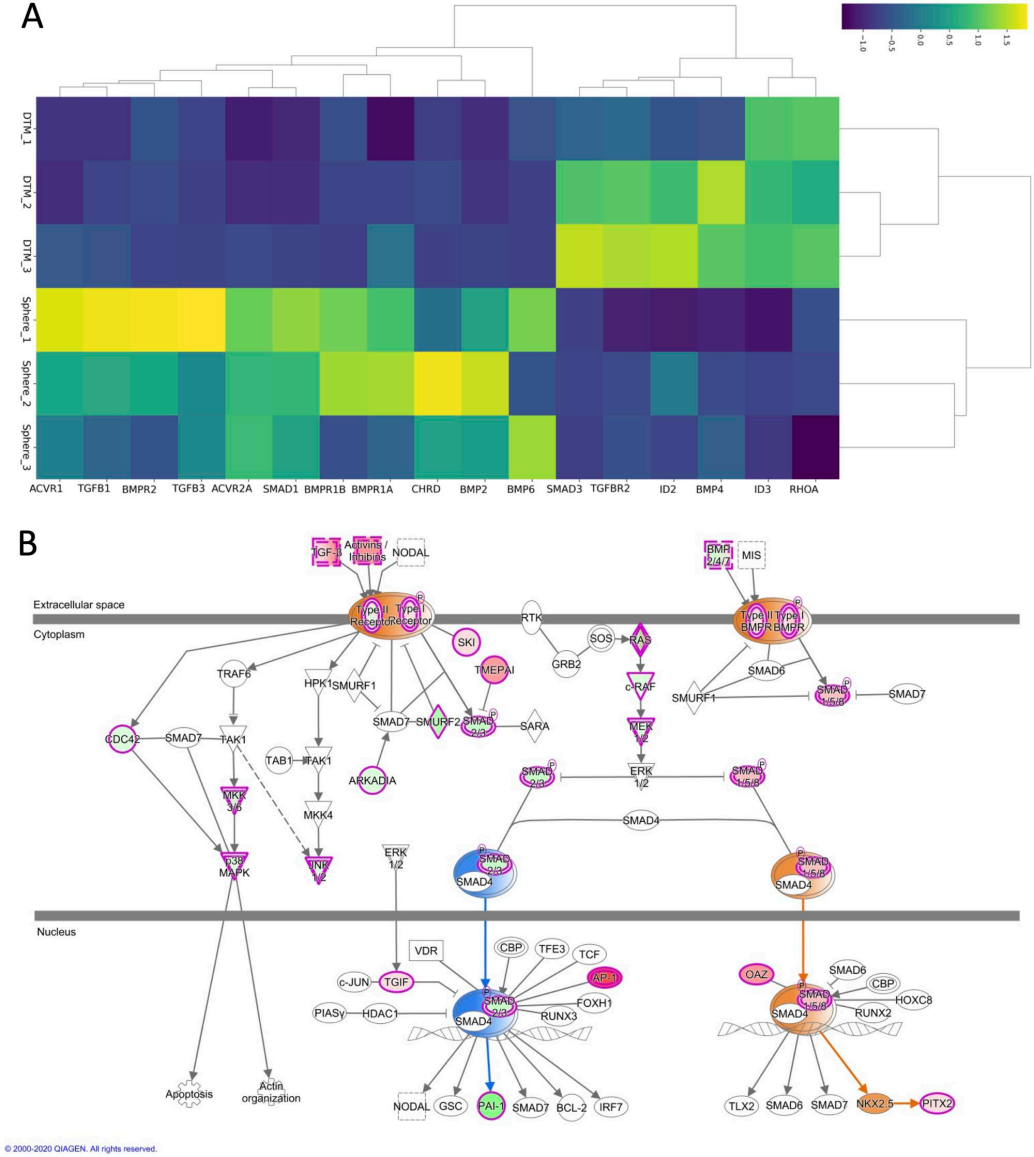
TGFβ–SMAD2/3 signaling is predicted to be inhibited in TMPCs relative to differentiated trabecular meshwork (TM) cells. (A) Heatmap showing the expression of differentiation-related DEGs associated with TGFβ signaling in TMPCs and differentiated TM cells. Lighter colors indicate higher expression levels. (B) Pathway map generated by IPA software, predicting inhibition of the SMAD2/3 pathway in TMPCs compared with TM cells. Node colors indicate gene expression and predict activity: red, upregulated DEGs in TMPCs; green, downregulated DEGs in TMPCs; orange, molecules predicted to be activated; blue, molecules predicted to be inhibited.

To identify pathways involved in TMPC differentiation, we performed pathway enrichment analysis using differentiation-associated DEGs between TMPCs and differentiated TM cells reported previously by our group.^18^ About 20% of the enriched differentiation-related pathways were linked to TGFβ signaling, with SMAD2/3-associated pathways showing the highest proportion of DEGs within their pathway-specific gene sets (Figures 1 and 2). Heatmap analysis of TGFβ signaling-related differentiation DEGs revealed an increase in SMAD3 and a decrease in SMAD1 expression in differentiated TM cells compared with TMPC spheres (Figure 3A). Pathway mapping further predicted inhibition of the SMAD2/3 signaling and activation of SMAD1/5/8 signaling in TMPCs relative to primary TM cells (Figure 3B). These findings suggest that the TGFβ-SMAD2/3 pathway is suppressed in TMPCs and becomes activated during their differentiation.

### Elevated TGFβ2 promotes differentiation of TMPCs into a fibrotic phenotype

**Figure 4.**
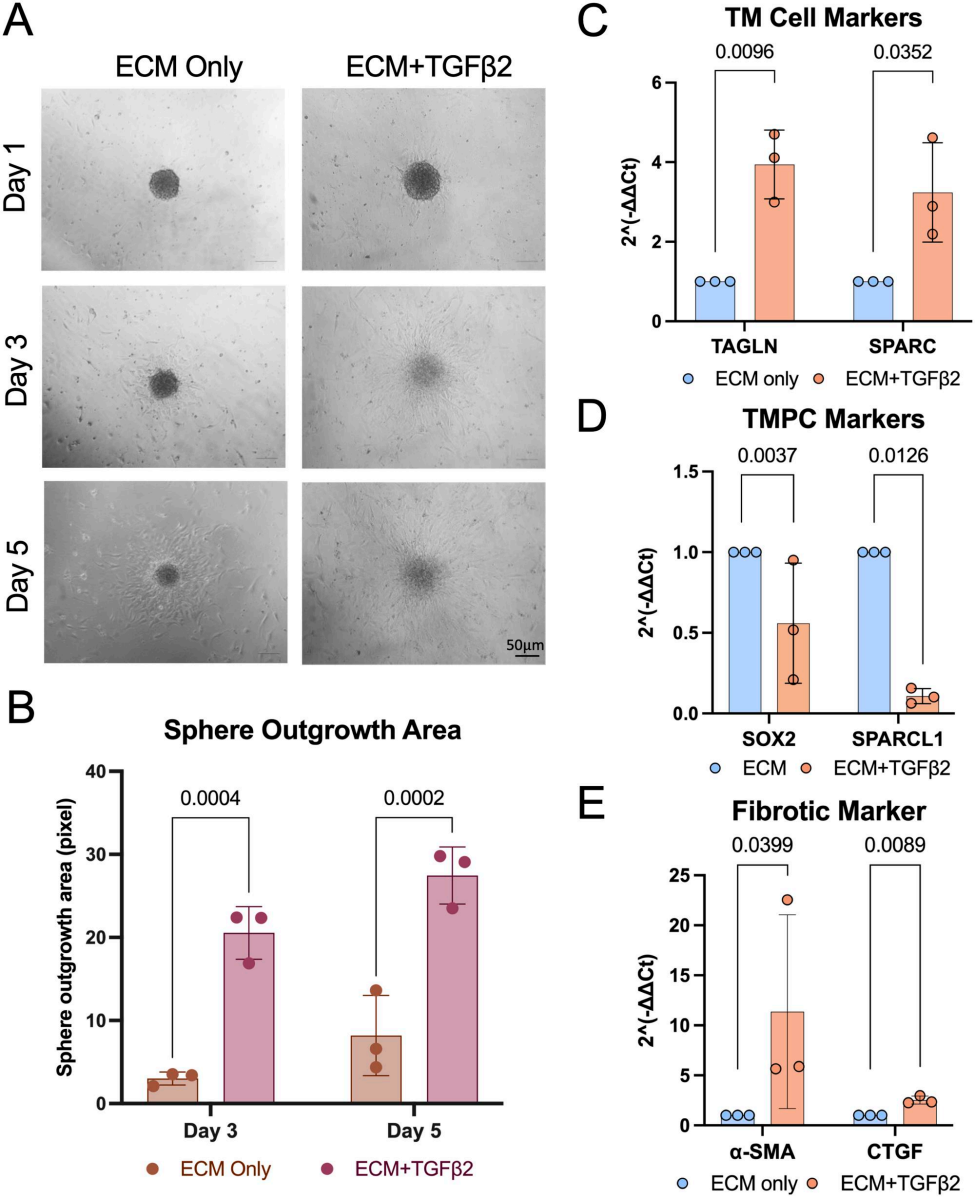
TGFβ2 treatment increases the mRNA differentiating TMPC spheres with/without TGFβ2 treatment on days 1, 3, and 5. Scale bar: 50 µm. (B) Cell expression of TM cell markers and decreases progenitor cell marker expression in TMPCs. (A) Phase contrast images of outgrowth from TMPC spheres was increased in the TGFβ2-treated group on days 3 and 5 (mean ± SD; *n* = 3 donors). Two-way ANOVA was used for statistical analyses. (C and D) RT-qPCR analysis showed upregulation of TM cell markers (SPARC and TAGLN), downregulation of TMPC markers (SOX2 and SPARCL1), and increased expression of fibrotic markers (α-SMA and CTGF). Data are presented as 2^ΔΔCT^ values (mean ± SD) from 3 donors (*n* = 3). Each gene was analyzed independently. Statistical analysis was performed on ΔΔCT values using a paired *t*-test (parametric) or Wilcoxon matched-pairs signed-rank test (non-parametric); *P* < .05 were considered significant.

**Figure 5.**
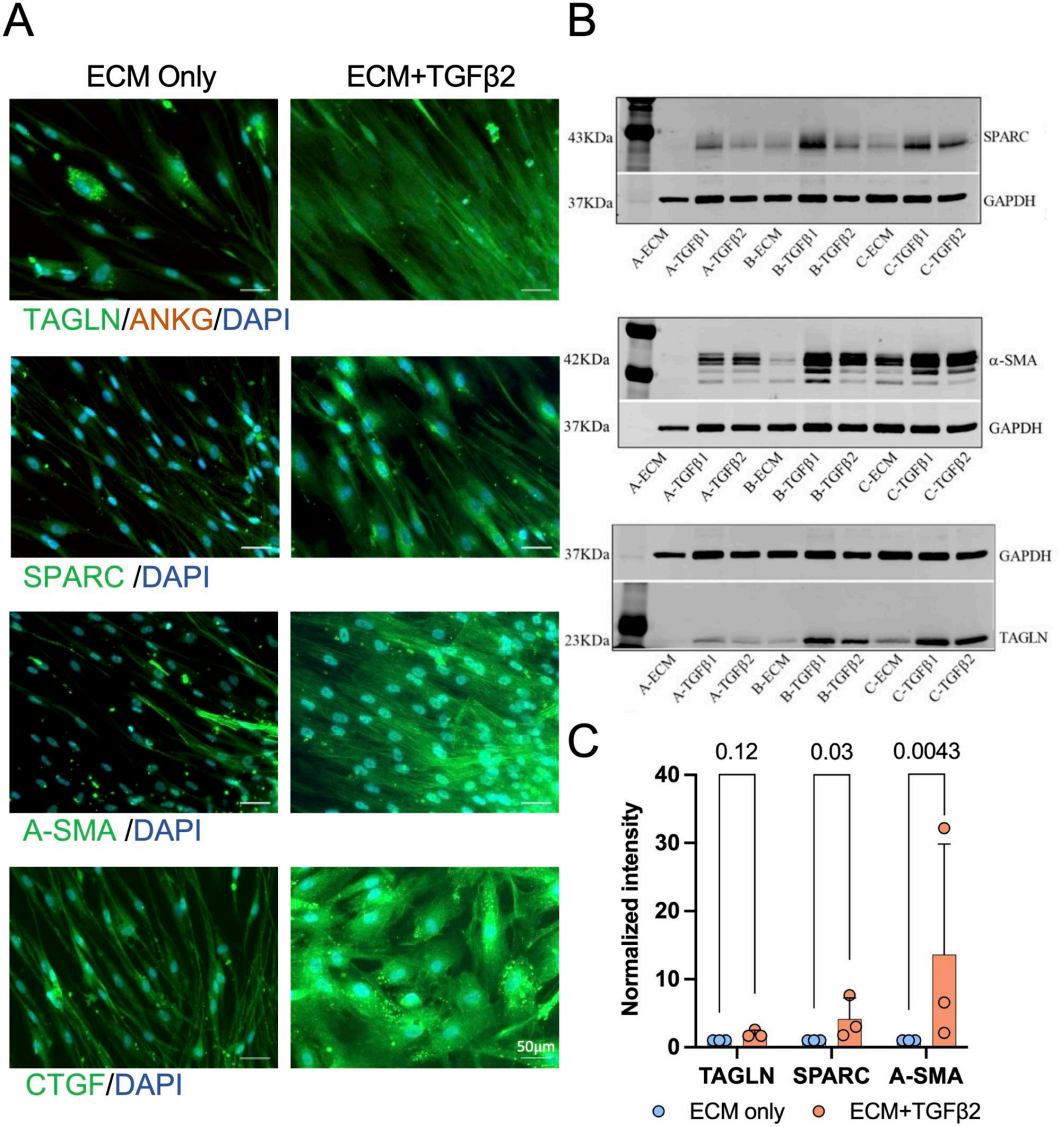
TGFβ2 treatment enhances the expression of differentiated TM cell markers and fibrotic markers in TMPCs. (A) Immunostaining showing increased expression of TM cell markers (TAGLN and SPARC) and fibrotic markers (α-SMA and CTGF) in TGFβ2-treated TMPCs compared with untreated controls. Experiments were replicated in 3 donors (*n* = 3). Scale bar, 50 µm. (B and C) Western blot analysis showing elevated protein levels of TAGLN, SPARC, and α-SMA following TGFβ2 treatment. Data are presented as staining intensity normalized to the untreated group (*n* = 3 donors). Each protein was analyzed independently. Paired *t*-test (parametric) or Wilcoxon matched-pairs signed-rank test (non-parametric) was used for comparisons. *P* < .05 was considered significant.

To examine the effect of elevated TGFβ2 on TMPC differentiation, TMPC spheres were treated with TGFβ2 at concentrations corresponding to those observed in the AH of POAG patients.^21^ From day 3 after TGFβ2 treatment, there was a significant increase of TMPC sphere cellular outgrowth in the TGFβ2-treated group compared with controls (Figure 4A and B). RT-qPCR showed upregulation of TM markers: transgelin (TAGLN) and osteonectin (SPARC),^18^^,^^37^ while progenitor markers, SRY (sex-determining region Y)-box transcription factor 2 (SOX2), and hevin (SPARCL1),^18^ were downregulated in the TGFβ2-treated group (Figure 4C and D). Immunostaining and western blotting confirmed increased TAGLN and SPARC at the protein level (Figure 5A and C). These results indicate that TGFβ2 drives TMPCs toward a differentiated TM cell phenotype.

In addition, expression of α-smooth muscle actin (α-SMA) and connective tissue growth factor (CTGF), canonical markers of fibroblastic transition previously associated with glaucomatous TM pathology,^38^^,^^39^ was upregulated in TGFβ2-treated cultures compared to controls both in transcript (Figure 4E) and protein levels (Figure 5A and C). These results demonstrate that TGFβ2 treatment induces upregulation of pro-fibrotic markers in TMPC-derived cells, consistent with acquisition of a fibroblast-like molecular profile.^40–43^

### Selective inhibition of SMAD2/3 reverses TGFβ2-induced differentiation in TMPCs

To determine whether inhibition of the SMAD2/3 pathway can prevent TGFβ2-induced TMPC differentiation, 2 selective small-molecule inhibitors were used: SB431542, which targets ALK5 (the receptor mediating SMAD2/3 activation), and ML347, which targets ALK1 (the receptor mediating SMAD1/5/8 activation, used here as a control). The effective concentrations and specificity of both inhibitors were confirmed in primary TM cells and adherent TMPCs. Upon TGFβ2 stimulation, nuclear translocation of p-SMAD2/3 and p-SMAD1/5/8 was observed, consistent with pathway activation, and was selectively blocked by 5 μM SB431542 or ML347 (Figure S1A–C).

**Figure 6.**
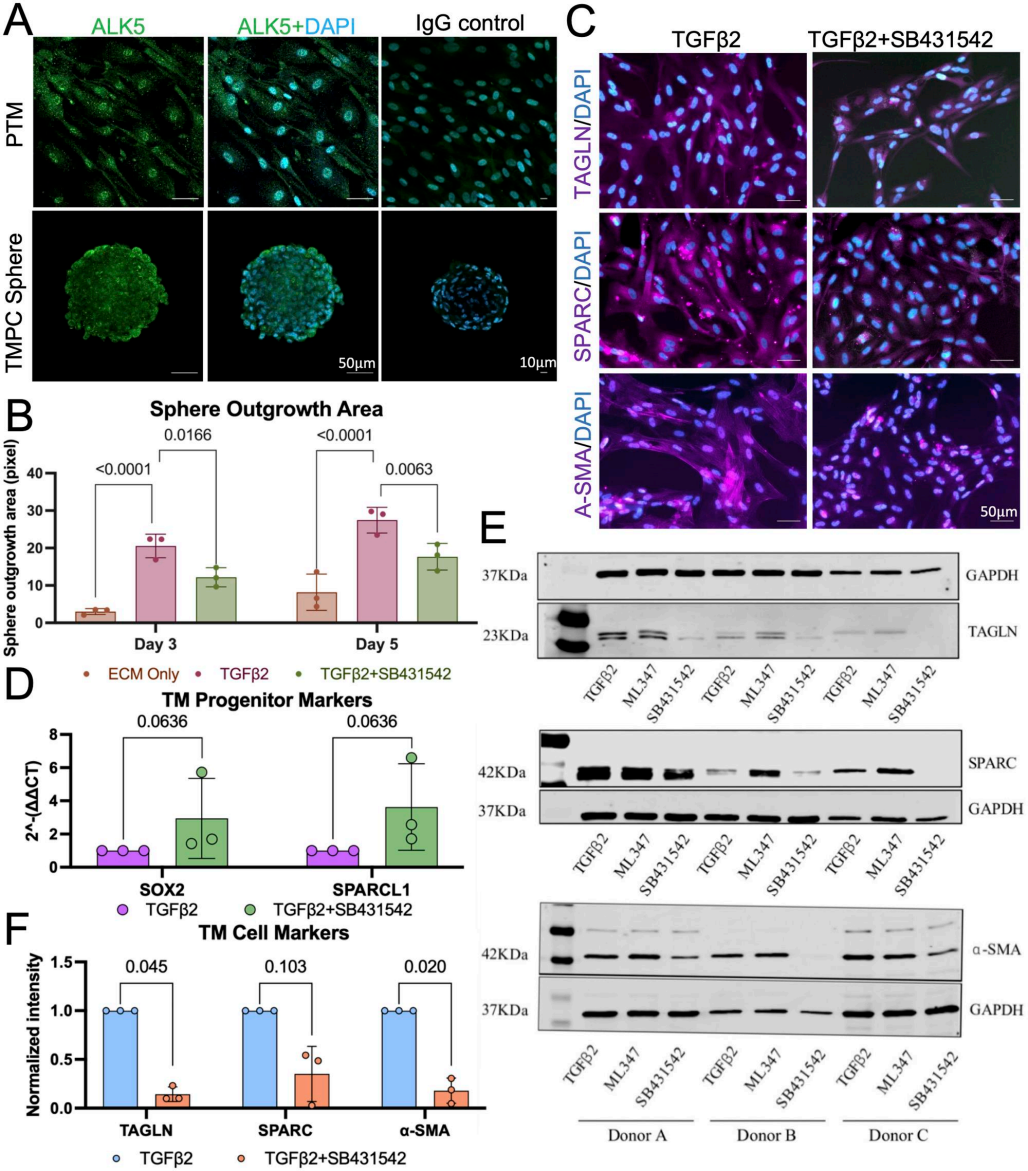
ALK5 inhibitor SB431542 treatment prevents TGFβ2-driven TMPC differentiation. (A) Immunostaining showed the SMAD2/3 pathway receptor ALK5 expressed in the TMPC spheres , Scale bar, 50 µm. IgG control scale bar: 10 µm. (B) TMPC sphere cell outgrowth area was decreased in SB431542 treatment group compared to TGFβ2 only group on day 5 (*n* = 3 donors). Two-way ANOVA was used for statistical analyses. (C) Immunostaining showed the decreased expression of TM cell markers TAGLN, SPARC and fibrotic marker α-SMA in SB431542 treatment group. Scale bar, 50 µm. （D）RT-qPCR showed the increased expression of TM progenitor cell markers SOX2 and SPARCL1 in SB431542 treatment group. Data are presented as 2^ΔΔCT^ values (mean ± SD) from 3 donors (*n* = 3). Each gene was analyzed independently. Statistical analysis was performed on ΔΔCT values using a paired *t*-test (parametric) or Wilcoxon matched-pairs signed-rank test (non-parametric). (E-F) Western blotting analyses showed significantly decreased expression of TM cell markers TAGLN, SPARC and fibrotic marker α-SMA in SB431542 treatment group (*n* = 3 donors). Each protein was analyzed independently. Paired *t*-test (parametric) or Wilcoxon matched-pairs signed-rank test (non-parametric) was used for statistical analyses. *P* < .05 was considered significant.

Immunostaining showed that the SMAD2/3 receptor ALK5 was expressed in TMPC spheres, with higher expression at the peripheral cell layers (Figure 6A). Treatment with the ALK5 inhibitor SB431542, followed by TGFβ2 exposure for 5 days, significantly reduced TMPC sphere outgrowth compared with TGFβ2 treatment alone (Figure 6B; Figure S2A). Inhibition of ALK5 decreased the expression of TM differentiation markers (TAGLN and SPARC) and fibrotic markers (α-SMA and CTGF), while restoring progenitor-associated markers (SOX2 and SPARCL1) at transcript and/or protein levels (Figure 6C–E). In contrast, inhibition of the ALK1-SMAD1/5/8 pathway with ML347 had minimal effects on the expression of these markers (Figure S2B–D). These results demonstrate that the blockade of the ALK5-SMAD2/3 pathway effectively suppresses TGFβ2-induced TMPC differentiation.

## Discussion

In this study, we identified TGFβ2-SMAD2/3 pathway as a key determinant of TMPC fate. Elevated TGFβ2 drives TMPCs toward a differentiated and fibrotic phenotype, accompanied by loss of progenitor identity. This mechanism provides new insight into POAG pathology. Inhibition of the ALK5-SMAD2/3 pathway blocks these effects, restoring progenitor marker expression and preventing fibrotic remodeling. These results highlight the ALK5–SMAD2/3 axis as a potential therapeutic target to preserve progenitor function and protect the TM in POAG.

In this study, we selected TAGLN and SPARC as markers of TM lineage commitment based on our previous findings demonstrating their differential expression between TMPCs and primary TM cells, supported by protein-level validation^18^ and confirmed expression in human TM tissue (unpublished data). These markers provided a robust framework for assessing differentiation toward a TM-like phenotype.

We also assessed SOX2 and SPARCL1 as markers of progenitor state. Both showed a trend toward increased expression following ALK5 inhibition; however, this did not reach statistical significance, likely due to the limited sample size. While informative, this targeted gene analysis is limited in its ability to fully capture the complexity of progenitor state. Further RNA sequencing would enable a more comprehensive and high-resolution assessment of global gene expression changes and cellular identity.

In this study, we also assessed MYOC due to its established association with TM biology and glaucoma. While MYOC has been reported to increase in primary TM cells following TGFβ2 stimulation,^44^ our previous RNA-seq data showed no significant difference between TMPCs and primary TM cells, and here its expression decreased following TGFβ2 treatment (data not shown). This suggests that MYOC regulation in TMPCs may differ from that of mature TM cells and is likely context dependent.

In this study, ECM derived from primary TM cells was employed as a biologically relevant scaffold to support TMPC differentiation and maintain structural integrity during culture. While the exact molecular composition of the isolated ECM was not defined, its use provided a stable and physiologically meaningful microenvironment to examine TMPC fate. Future studies integrating extended culture systems with detailed molecular and proteomic profiling of ECM components will further clarify how matrix–cell interactions influence progenitor maintenance and fibrotic transition within the TM niche.

To define the phenotypic state of differentiated TMPCs, we examined established fibrotic markers, including α-SMA and CTGF. TGFβ2 treatment significantly increased expression of both markers at the transcript and protein levels, consistent with prior evidence demonstrating that TGFβ2 promotes fibrotic remodeling in TM cells and tissues.^31^^,^^38^^,^^45^^,^^46^ α-SMA, a key regulator of cellular contractility and ECM remodeling,^47^ is elevated in glaucomatous TM and ocular hypertension models,^45^^,^^46^^,^^48^ while CTGF has been implicated in TM dysfunction and IOP elevation.^38^^,^^49^^,^^50^ Together, these findings indicate that TGFβ2 exposure induces a pro-fibrotic molecular program in TMPC-derived cells. However, it remains unclear whether this fibrotic phenotype arises during TMPC differentiation or reflects changes that occur after differentiation into mature TM cells. Furthermore, the relative contribution of TMPC-specific alterations versus fibrosis in the broader TM cell population to the POAG-associated phenotype remains to be determined. Future studies incorporating side-by-side comparisons with validated fibrotic TM models, along with functional assessments of matrix remodeling, cellular contractility, and tissue biomechanics, will help to better define the myo-fibrotic phenotype and clarify its cellular origin.

We also included TGFβ1 as a comparative stimulus to better delineate isoform-specific effects. Although TGFβ1 and TGFβ2 share structural similarity and overlapping functions, they are associated with distinct glaucoma subtypes. TGFβ2 is more consistently elevated in the AH of patients with POAG, whereas TGFβ1 has been more frequently linked to pseudoexfoliative glaucoma.^51^ In our system, both isoforms elicited comparable effects on TM lineage markers (TAGLN and SPARC) and α-SMA expression, indicating shared pro-differentiation capacity. Nevertheless, we centered our mechanistic analyses on TGFβ2 to more closely model the molecular milieu relevant to POAG. Ongoing studies examining TGFβ1 responses will further refine understanding of isoform-specific regulatory mechanisms.

To clarify the signaling mechanisms driving this phenotype, we used selective inhibitors of ALK1 (ML347) and ALK5 (SB431542).^52^^,^^53^ ALK5 inhibition with SB431542 suppressed TGFβ2-induced SMAD2/3 activity and reversed fibrotic marker expression, including α-SMA and CTGF. In contrast, ALK1 inhibition with ML347 did not significantly impact differentiation outcomes. Cells treated with SB431542 retained a more progenitor-like morphology and expressed lower levels of TM and fibrotic markers suggesting that ALK5–SMAD2/3 signaling is the dominant driver of TMPC differentiation and fibrotic phenotype in this context. This is consistent with prior findings that SMAD2/3 signaling promotes stem cell differentiation, while SMAD1/5 activity supports stem cell maintenance.^23^^,^^30^ Additionally, SB431542 has been shown to reduce TM stiffness and lower IOP in vivo,^54^ supporting its therapeutic relevance.

While our findings delineate a clear molecular mechanism by which TGFβ2 influences TMPC fate in vitro, further studies will be important to extend these observations to functional and physiological contexts. Integrating analyses of ECM remodeling, tissue mechanics, and AH outflow dynamics will strengthen understanding of how these molecular changes translate to tissue-level outcomes. Although primary human donor samples inherently limit experimental scale, the consistency observed across donors supports the robustness and biological relevance of the findings. Moreover, while ALK inhibition reduced TM markers and increased SOX2 and SPARCL1 expression, broader transcriptomic profiling will be valuable to comprehensively assess progenitor state preservation.

It is also important to note that SB431542 targets ALK4 and ALK7 in addition to ALK5. As these receptors converge on SMAD2/3 activation, pharmacological inhibition suppresses downstream signaling broadly within this axis. Complementary genetic approaches will help refine receptor-specific contributions and further clarify mechanistic specificity.

Beyond the ALK5–SMAD2/3 pathway, our analysis identified several additional signaling pathways involved in TMPC differentiation, including BMP, RAC1, estrogen receptor, EPHB, and β-catenin–related signaling. These pathways are known to play important roles in controlling stem cell behavior and lineage commitment. For example, BMP signaling was reported to promote mesenchymal differentiation.^55^ Cell adhesion–related pathways, such as NrCAM and Eph receptors, may influence differentiation by modulating cell–cell and cell–matrix interactions, including effects on β-catenin activity.^56^^,^^57^ Estrogen receptor signaling has also been shown to affect progenitor cell fate through multiple mechanisms.^58^ Enrichment of these pathways in our bioinformatic analysis suggests that TMPC differentiation involves integration of multiple signaling axes beyond the core TGF-β–SMAD2/3 circuit, reflecting a complex network of extracellular and intracellular cues that guide stem cell commitment.

**Figure 7.**
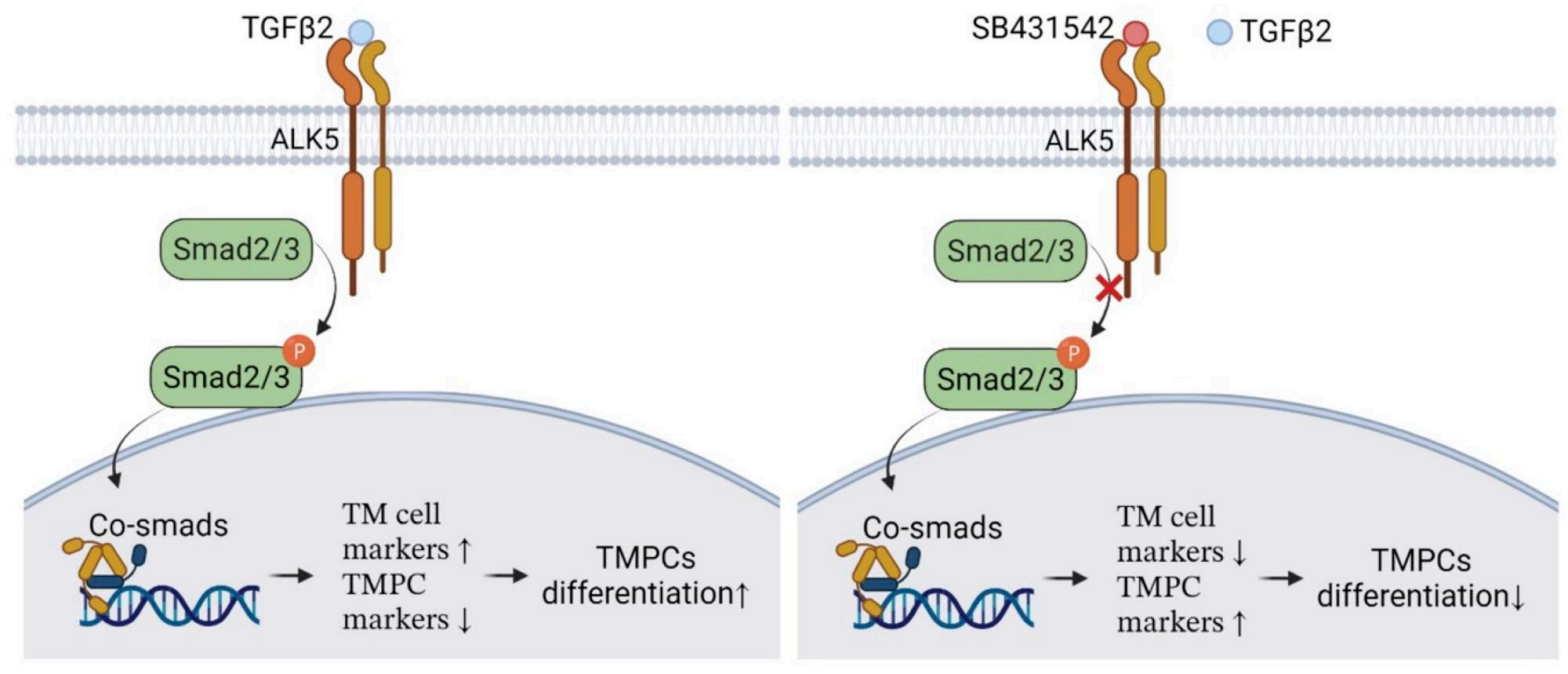
Schematic diagram summary of the promotion effect of TGFβ2 on the TM progenitor cell differentiation through TGFβ2-SMAD2/3 pathway.

In conclusion, our study identifies TGFβ2 as a central regulator of TMPC fate, promoting differentiation and a pro-fibrotic transition through activation of the SMAD2/3 pathway in vitro. Inhibition of ALK5 attenuated these responses, partially maintaining progenitor-associated characteristics and reducing induction of fibrotic genes, thereby delineating a TGFβ2–ALK5–SMAD2/3 signaling axis that shapes TMPC phenotypic commitment (Figure 7). While these findings provide direct mechanistic insight at the cellular level, their relevance to IOP regulation and POAG pathogenesis remains to be established in physiologically relevant in vivo and disease models. Together, these findings provide a mechanistic framework for understanding how sustained TGFβ2 signaling influences TM progenitor dynamics and lay the groundwork for future studies aimed at translating this axis into targeted therapeutic strategies for glaucoma.

## Supplementary Material

sxag033_Supplementary_Data

## Data Availability

The datasets generated and analyzed during the current study are available from the corresponding authors.
