# Supplementary material for: Transforming growth factor beta-2 drives trabecular meshwork progenitor cell differentiation through SMAD2/3 signaling
Source: Stem Cells. 2026 May 26;44(8):sxag033. doi: 10.1093/stmcls/sxag033 (PMC13446226; doi:10.1093/stmcls/sxag033)
Supplement: Supplementary file 1 [file sxag033_supplementary_data.zip › Supplementary Information-R4.docx]

**Supplementary Information**

**Running title: TGFβ2 Drives TM Cell Differentiation**

**Transforming Growth Factor beta-2 (TGFβ2) Drives Trabecular Meshwork Progenitor Cell Differentiation Through SMAD2/3 Signalling**

Xiaochen Fan ^1,4*^, Emine K. Bilir ^1,2^, Olivia Kingston ^1^, Victoria R. Kearns ^1^, Colin E. Willoughby ^1,3*^, Carl M. Sheridan ^1*^

^1^ Department of Eye and Vision Science, Institute of Life Course and Medical Sciences, University of Liverpool, Liverpool L69 3BX, UK.

^2^ Department of Musculoskeletal and Ageing Science, Institute of Life Course and Medical Sciences, University of Liverpool, Liverpool L69 3BX, UK.

^3^ Centre for Genomic Medicine, Biomedical Sciences Research Institute, Ulster University, Coleraine BT52 1SA, UK.

^4^ Institute of Ophthalmology, University College London, London, EC1V 9EL, UK

*Corresponding authors

Contact information:

[c.sheridan@liverpool.ac.uk](mailto:c.sheridan@liverpool.ac.uk)

Department of Eye and Vision Science,

Institute of Life Course and Medical Sciences,

University of Liverpool,

6 W Derby St,

Liverpool,

L7 8TX,

United Kingdom.

**Supplementary Tables**

**Supplementary Table S1: Information of the donors used for primary TM cell and TM sphere culture**

| Donor ID | Age | Eye related disease |
| --- | --- | --- |
| HCR020 | 75 | None |
| HCR030 | 21 | None |
| NHS038 | 81 | None |

Supplementary table S1 shows the information of the donors used in this experiment that include the donor ID, age and eye related disease.

**Supplementary Table S2: Information of the primary antibodies used in immunocytochemistry**

| Primary Antibodies | Company | Catalogue | Species | Dilution |
| --- | --- | --- | --- | --- |
| P-SMAD2/3 | Cell Signaling | 8685s | Rabbit | 1:100 |
| Myocilin | R&D Systems | MAB3446 | Mouse | 1:10 |
| SPARC | Proteintech | 15274-1-AP | Rabbit | 1:50 |
| TAGLN | Thermo Fisher | PA513632 | Rabbit | 1:50 |
| α-SMA | Abcam | Ab5694 | Rabbit | 1:100 |
| ANKG | ThermoFisher | 33-800 | Mouse | 1:250 |
| ALK1 | Abcam | Ab68703 | Rabbit | 1:100 |
| ALK5 | Abcam | Ab31013 | Rabbit | 1:100 |
| IgG mouse | Thermo Fisher | 10400C | Mouse | 1:600 |
| IgG rabbit | Thermo Fisher | 10500C | Rabbit | 1:600 |

### Supplementary table S2 shows the information of the primary antibodies used in the Immunocytochemistry (name, company, catalogue number, species and dilution).

**Supplementary Table S3: Information of the secondary antibodies used in immunocytochemistry**

| Secondary Antibodies | Company | Catalogue | Dilution |
| --- | --- | --- | --- |
| Goat anti-Rabbit, Alexa Fluor Plus 488 | Thermo Fisher | A-11008 | 1:500 |
| Goat anti-mouse, Alexa Fluor Plus 594 | Thermo Fisher | A-11005 | 1:500 |

### Supplementary table S3 shows the information of the secondary antibodies used in the Immunocytochemistry (name, company, catalogue number and dilution).

**Supplementary Table S4: Information of primary antibodies used in western blotting**

| Primary antibodies | Company | Catalogue | Species | Dilution |
| --- | --- | --- | --- | --- |
| α-SMA | Abcam | Ab5694 | Rabbit | 1:200 |
| SPARC | Proteintech | 15274-1-AP | Rabbit | 1:500 |
| TAGLN | Thermo Fisher | PA513632 | Rabbit | 1:500 |
| GAPDH | Abcam | ab8245 | mouse | 2 mg/mL |

Supplementary table S4 shows the name, company, catalogue number, species and dilution information of the primary antibodies used in western blotting.

**Supplementary Table S5: Information of secondary antibodies used in western blotting**

| Secondary antibodies | Company | Catalog | Dilution |
| --- | --- | --- | --- |
| IRDye 680RD  Goat anti-Rabbit | LI-COR | C80816-15 | 1:15000 |
| IRDye 800CW  Goat anti-Mouse | LI-COR | C80605-15 | 1:15000 |

Supplementary table S5 shows the name, company, catalogue number and dilution information of the secondary antibodies used in western blotting

**Supplementary Table S6: Information of primer sequences**

| Gene | Forward primers (5’-3’) | Reverse primers (5’-3’) | Melting T (°C) |
| --- | --- | --- | --- |
| *ABCA1* | AACAGTTTGTGGCCCTTTTG | AGTTCCAGGCTGGGGTACTT | 60 |
| *ACTA2* | CCAGAGCCATTGTCACACAC | CAGCCAAGCACTGTCAGG | 60 |
| *ALK1* | CGGACTGCTTTGAGTCCTACA | AGTCCTCCACGATGCCATTC | 60 |
| *ALK5* | CGACGGCGTTACAGTGTTTCT | CCCATCTGTCACACAAGTAAA | 60 |
| *ANKG* | GAAGATGCAATGACCGGGGA | CTGAAGCCCATGTAACCCTCTG | 60 |
| *APOE* | TTCCCCAGGAGCCGACTG | GAATGTGACCAGCAACGCAG | 52 |
| *CHOP* | AAGGCACTGAGCGTATCATGT | TGAAGATACACTTCCTTCTTGAACA | 50 |
| *CTGF* | GAAGAGAACATTAAGAAGGGCA | TCCACAGAATTTAGCTCGGT | 60 |
| *FN1* | TGGTATTCAGCTTCCTGGCA | CGGGTATGGTCTTGGCCTAT | 60 |
| *FOXC1* | CCACTCGGTGCGGGAAATG | ACGTGCGGTACAGAGACTGA | 60 |
| *GAPDH* | CGAGCCACATCGCTCAGACACC | GGTCAATGAAGGGGTCATTGATGGCAAC | 60 |
| *GRP78* | TGGGTCGACTCGAATTCCAAAG | GTCAGGCGATTCTGGTCATTGG | 50 |
| *IL6* | GGTACATCCTCGACGGCATCT | GTGCCTCTTTGCTGCTTTCAC | 52 |
| *Myocilin* | AAGCCCACCTACCCCTACAC | TCCAGTGGCCTAGGCAGTAT | 60 |
| *PDGFRA* | TACTGTTGGAGCTACAGGGA | TCTCCTCCGATGTTATTCCG | 60 |
| *SOX2* | CCCACCTACAGCATGTCCTACTC | TGGAGTGGGAGGAAGAGGTAAC | 60 |
| *SPARC* | CTGGACTACATCGGGCCTTG | ATGGATCTTCTTCACCCGCAG | 60 |
| *SPARCL1* | GCTGCAATCCCGACAAATGC | AGTGTTGTCAGGTGCTACCG | 60 |
| *TAGLN* | AAGCCTTCTTTCCCCAGACA | TGCACTATGATCCACTCCACC | 60 |

Supplementary table S6 shows the primer sequences and melting temperature of the primer pairs used in the RT-qPCR analyses.

**Supplementary Table S7: RT-qPCR running conditions**

| Step | Cycle | Temperature | Time  (mm:ss) | Ramp Rate (°C/s) |
| --- | --- | --- | --- | --- |
| Denaturation | 1 | 95 | 05:00 | 4.4 |
| Amplification | 45 | 95 | 00:10 | 4.4 |
|  |  | 60 | 00:10 | 2.2 |
|  |  | 72 | 00:12 | 4.4 |
| Melt Curve | 1 | 95 | 00:05 | 4.4 |
|  |  | See Table 6 | 01:00 | 2.2 |
|  |  | 97 | Continuous | 0.11 |
| Cooling | 1 | 40 | 00:30 | 2.2 |

Supplementary table S7 shows the RT-qPCR running conditions including the different steps, cycle numbers, temperature, times and ramp rates.

**Supplementary Table S8: Differentiation-related GO terms enriched in the DEGs (TMPCs vs differentiated TM cells)**

| GO Term ID | Description | Gene Ratio | p.adjust | For pathway analysis |
| --- | --- | --- | --- | --- |
| GO:0045666 | positive regulation of neuron differentiation | 129/4130 | 1.099826e-06 | Y |
| GO:0002062 | chondrocyte differentiation | 43/4130 | 1.275366e-04 | Y |
| GO:0001649 | osteoblast differentiation | 78/4130 | 7.189248e-04 | Y |
| GO:0048762 | mesenchymal cell differentiation | 70/4130 | 1.471034e-03 | Y |
| GO:0032330 | regulation of chondrocyte differentiation | 22/4130 | 3.449098e-03 |  |
| GO:0010001 | glial cell differentiation | 71/4130 | 4.967490e-03 |  |
| GO:0051146 | striated muscle cell differentiation | 85/4130 | 5.178159e-03 |  |
| GO:0002244 | hematopoietic progenitor cell differentiation | 55/4130 | 7.144904e-03 |  |
| GO:0045598 | regulation of fat cell differentiation | 47/4130 | 8.078371e-03 |  |
| GO:0045667 | regulation of osteoblast differentiation | 46/4130 | 8.658417e-03 |  |
| GO:0042692 | muscle cell differentiation | 109/4130 | 9.928869e-03 |  |

Supplementary table S8 shows the differentiation-related GO terms enriched in the DEGs (TMPCs vs differentiated TM cells) with their ID, description, gene ratio and adjusted p-value. Y: Differentiation-related GO terms used for further pathway analyses.

**Supplementary Figure Legends**

**Supplementary Figure S1. Evaluation of ALK inhibitor efficacy in modulating SMAD2/3 and SMAD1/5/8 signalling pathways. (A-B)** Immunostaining showed nuclear translocation of p-SMAD2/3 and p-SMAD1/5/8 staining (grey) in primary TM cells (PTM) or TMPCs after TGFβ2 treatment for 30min (p-SMAD2/3) or 24h (p-SMAD1/5/8). Yellow arrows indicate cytoplasmic localisation of p-SMADs in untreated TM cells, which was reduced upon TGFβ2 treatment. **(C)** The ALK5 inhibitor SB431542 (2-20 µM) effectively suppressed TGFβ2-induced nuclear translocation of p-SMAD2/3 in PTM cells, whereas the ALK1 inhibitor ML347 (2-20 µM) had no observable effect. **(D)** Conversely, ML347 (2-10 µM), but not SB431542, inhibited TGFβ2-induced nuclear translocation of p-SMAD1/5/8 in adherent TMPCs. Scale bar, 50µm.

**Supplementary Figure S2. Inhibition of the SMAD1/5/8 pathway does not rescue TGFβ2-induced differentiation of TMPCs. (A)** Phase-contrast images of TMPC spheres treated with TGFβ2 in the presence or absence of ALK inhibitors. Black arrows indicate visible TMPC spheres. **(B)** RT-qPCR analysis showed no significant changes of TM cell markers (SPARC, TAGLN), TMPC markers (SOX2, SPARCL1), and fibrotic markers (α-SMA, CTGF). Data are presented as 2^ΔΔCT values (mean±SD) from three donors (n=3). Statistical analysis was performed on ΔΔCT values using a paired t-test. **(C)** Immunostaining showed no detectable differences in TAGLN, SPARC, or α-SMA expression (purple) between TGFβ2 ± ML347 treatment groups. **(D)** Quantitative analysis of western blotting confirmed the absence of significant changes in TAGLN, SPARC, and α-SMA protein levels between TGFβ2 ± ML347 conditions. Scale bar, 50µm. P < 0.05 were considered significant.

**Supplementary Figure S3. Characterisation of trabecular meshwork (TM) cells based on myocilin induction following dexamethasone (DEX) treatment. (A, D)** Immunofluorescence staining of myocilin (green) in primary TM cells derived from three donors (NHS033, NHS038, and NHS050) used in the present study, as well as in samples previously subjected to RNA-sequencing for bioinformatic analyses. Scale bars: 20μm (A) and 100μm (D). **(B, C)** Western blot analyses demonstrate increased myocilin protein expression in TM cells (NHS033, NHS038, and HCR030) following treatment with 100nM DEX for 7 days. **(E)** RT–qPCR analysis shows a corresponding upregulation of myocilin (MYOC) mRNA levels in TM cells (NHCR013, HCR020, and HCR030) after 7 days of treatment with 100nM DEX.
